# Study of the Formation and Solution Properties of Worm-Like Micelles Formed Using Both *N*-Hexadecyl-*N*-Methylpiperidinium Bromide-Based Cationic Surfactant and Anionic Surfactant

**DOI:** 10.1371/journal.pone.0110155

**Published:** 2014-10-08

**Authors:** Zhihu Yan, Caili Dai, Haishun Feng, Yifei Liu, Shilu Wang

**Affiliations:** School of Petroleum Engineering, China University of Petroleum (Huadong), Qingdao, P. R. China; Texas A&M University, United States of America

## Abstract

The viscoelastic properties of worm-like micelles formed by mixing the cationic surfactant *N*-hexadecyl-*N*-methylpiperidinium bromide (C_16_MDB) with the anionic surfactant sodium laurate (SL) in aqueous solutions were investigated using rheological measurements. The effects of sodium laurate and temperature on the worm-like micelles and the mechanism of the observed shear thinning phenomenon and pseudoplastic behavior were systematically investigated. Additionally, cryogenic transmission electron microscopy images further ascertained existence of entangled worm-like micelles.

## Introduction

It is well known that amphiphilic surfactants exhibit an impressive range of polymorphic self-aggregates in solution, such as micelles, micro emulsions, liquid crystals and vesicles [1]–[4]. Among these organized aggregate structures, worm-like micelles are spontaneously formed entangled networks that impart excellent viscoelastic properties to the surfactant solutions similar to those of flexible polymer solutions. However, unlike flexible polymers that are connected by strongly covalent bonds, worm-like micelles are held together by weaker intermolecular forces that can reversibly break and recombine easily. Owing to this unique characteristic, over the past two decades, worm-like micelles have become popular in many applications, such as fracturing fluids, heat-conducting fluids, and personal care products [5]–[7].

An amphiphilic molecule typically consists of a head group possesses hydrophilic properties and a tail that has hydrophobic properties. If the head group is ionic, the molecule becomes charged in aqueous solution through the ionization effect. The molecule remains uncharged if the head group is nonionic, instead possessing characteristics of favoring the aqueous environment due to high polarity. Zwitterionic molecules contain head groups with two charges with opposite signs. It is common that the hydrophobic tail is a hydrocarbon, though fluorocarbons are also seen. In some cases, the hydrophobic region consists of two tails (e.g. biological lipids). When above the critical micelle concentration, amphiphilic surfactants in water have the tendency to self-assemble to form spherical aggregates, in which the outer layer is composed of hydrophilic head groups joined by intermolecular forces and the hydrophobic tails are sequestered internally. From a physical chemistry point of view, the formation of spherical aggregates is a common phenomenon for amphiphilic surfactants. Early in spherical aggregate formation, the viscosity of the surfactant solution is only slightly greater than water and is similar to a Newtonian fluid. However, under appropriate conditions of surfactant concentration, salinity and temperature, the structure transition from spherical micelles to long and flexible micelles is a crucial step that brings about the highly viscoelastic behavior in aqueous solutions. The formation of different types of worm-like micelles (e.g. cationic/anionic and ionic/nonionic) have been previously studied.

Ionic liquids (ILs) are also known as organic molten electrolytes. Among them, the surface active ILs (SAILs) are interesting because of their unusual physicochemical properties, such as their catalytic properties, high conductivity, negligible vapor pressure, and non-flammability [8]. The great advantage of SAILs, which have a hydrophobic chain and a hydrophilic head group, is their structural designability via the interchange their cations or anions. Zhao and Chen formed a novel gel phase in aqueous solutions by mixing *1*-hexadecyl-*3*-methylimidazolium chloride (C_16_mimCl) and SDS [9]. Zhao and Zheng synthesized *N-*alkyl-*N-*methylpyrrolidinium bromide (C_n_MPBr) and studied its multiple assembly behaviors [10]. Varade and co-workers studied the micellization and structure of cetylpyridinium chloride (CPyCl) micelles [11].

Thus far, much of the research focus has been on SAILs with imidazolium, pyrrolidinium and pyridinium head groups. In this contribution, we explore the worm-like micelles formed by *N*-alkyl-*N*- methylpiperidinium bromide (C_16_MDB) in the presence of sodium laurate (SL) in aqueous solutions. Rheometer measurements demonstrated that worm-like micelles solutions showed high viscoelasticity with relaxation behavior, in accordance with a Maxwell model with a single relaxation time. To conclusively establish the microstructure of the worm-like micelles, cryogenic transmission electron microscopy (cryo-TEM) was utilized to directly visualize worm-like micelles. The mechanism of the observed shear thinning phenomenon and pseudoplastic behavior were systematically investigated. In addition, the rheological parameters were calculated, and the influenced of temperature on the morphology of worm-like micelles was discussed.

## Materials and Methods

### Materials

**Figure 1:**
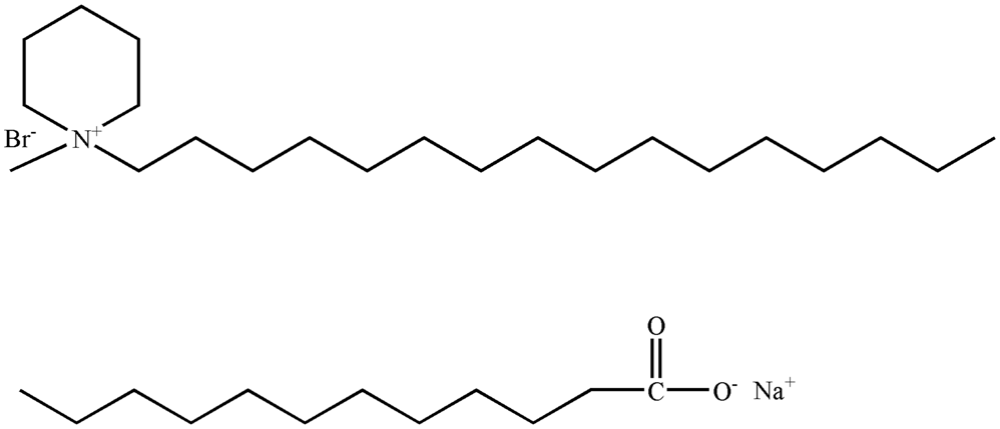
Chemical structure of C_16_MDB (a) and Sodium Laurate (b).

The schematic chemical structures of C_16_MDB and SL are shown in Figure 1. Cationic surfactant C_16_MDB was synthesized and purified according to a published method [12]. Anionic surfactant SL was an AR grade product of the Aladdin Chemistry Company and was used without purification. All of the aqueous solutions were prepared with distilled-deionized water.

### Sample Preparation

The samples were prepared by simply dissolving SL in C_16_MDB stock solutions. The cationic surfactant concentration of C_16_MDB was fixed at 70 mM. The anionic surfactant SL was at a molar concentration ranging from 20 mM to 70 mM. The samples were homogenized by mild heating and vortex mixing. Before any rheological measurements, all samples were stored in a thermostatic bath at 25°C for at least a week to equilibrate.

### Rheological measurements

The rheological property measurements were performed on a Physica MCR301 rheometer made by Anton Paar GmbH with a Rotor CC27 system. The shearing rate ranged from 0.01 to 1000 s^−1^ in the steady shear experiment. For the dynamic oscillatory measurements, dynamic strain sweep measurements were employed to confirm the linear viscoelastic region, and the frequency region was set to a range of 0.01–100 rad s^−1^. To guarantee experimental accuracy with regard to temperature effects, the samples were allowed to stabilize for at least 20 min before each rheological test.

### Cryo-TEM

Cryo-TEM measurements were performed with a Gatan cryo Holder 626 and a FEI Tecnai 20 TEM (200 kV) at approximately −174°C. The images were captured with a Gatan US1000 894 CCD and processed with Leginon software. To maintain the structural integrity of the worm-like micelles, Cryo-TEM samples were prepared in a controlled environment chamber to avoid damage and contamination during sample preparation. The samples were prepared with the following sequence of operations. Five milliliters of the sample was placed onto a perforated polymer film held by tweezers to ensure that the formation of the thin film spanned the mesh hole. After 10 seconds, the samples were immediately immersed into liquid ethane cooled by the nitrogen below its freezing point of −183°C. The samples were then stored in liquid nitrogen to protect against contamination before examination. Several images of each sample were taken, and a representative image was chosen to present.

### Theoretical Considerations

The Maxwell model was used to describe the viscoelasticity and relaxation behavior of the worm-like micelles. The elastic modulus (storage modulus) *G′* and the viscous modulus (loss modulus) *G″* are given by [1], [13]:
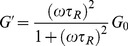
(1)

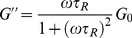
(2)Here, *ω* is the frequency, 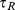 is the relaxation time and is approximately equal to the reciprocal of 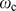, 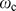 is the crossover frequency when *G′* and *G″* intersect, and *G_0_* is the plateau modulus, which is measured by reaching a plateau at high frequency.

## Results and Discussion

### Steady-State Rheological Results

**Figure 2:**
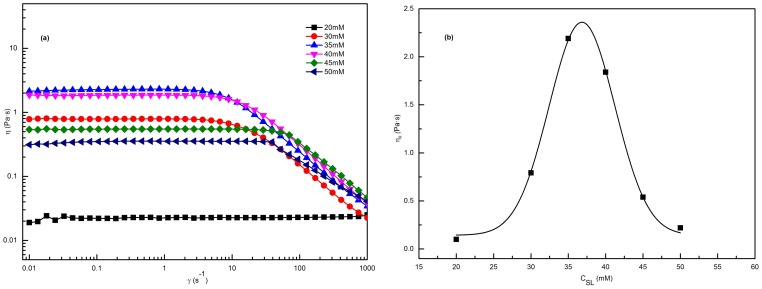
Steady rheology plots. (a) Steady rheology plots for 70 mM C_16_MDB with different SL concentrations at *T* = 25°C; (b) Variations of zero-shear viscosity (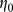) as a function of different SL concentrations for the 70 mM C_16_MDB.

Figure 2a represents the steady shear viscosity as a function of the shear rate for micelles prepared using various concentrations of SL. At low concentrations of SL, the viscosity of the sample is independent of shear, which conforms with the expected behavior of a Newtonian fluid. When the concentration reached 30 mM, the phenomenon of shear thinning was observed at higher shear rates. Shear thinning is a typical behavior of worm-like micelles and is thought to be the deformation and alignment of worm-like micelles with the entangled network due to the action of the applied orienting forces [14]. As the concentration of SL is increased further, the critical shear rate decreases firstly, and then increases. This observation can be attributed to the structural differences of the formed worm-like micelles. When the concentration of the SL increases beyond 50 mM, it is interesting to note that the solution separates spontaneously into two immiscible aqueous phases when left undisturbed. There is a clear interfacial boundary between the upper phase and lower phase and almost no color difference. This phenomenon will reappear even if the solution is vortexed several times. This dual phase behavior is commonly known as “aqueous surfactant two-phases” (ASTP) and usually appears in certain mixed anionic-cationic surfactant systems [15]. It is quite clear that ASTP is different from the phase demixing observed in solutions in which a single surfactant dissolved. The main factors that influence the formation of ASTP are the molar ratios of the two surfactants and the total concentrations of the system. When the concentration of SL exceeded 60 mM, the color of the solutions changed from transparent to milky. The reason for this phenomenon is the formation of precipitate that is triggered by strong interactions between the cationic and anionic head groups in surfactant mixtures close to equimolar composition, which leads to charge neutralization and subsequent decrease of aqueous solubility [16].

Figure 2b illustrates the variation of the zero-shear viscosity (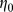) as a function of the SL concentration. 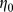 was calculated from the average viscosity at the low shear rate. Initially, when the concentration of SL was very low, there was almost no change in 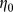. When the concentration of SL was greater than 30 mM, 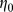 grew rapidly and reached a maximum at 35 mM SL. Above this concentration, 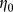 decreased. It is commonly believed that variations in 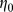 reflect changes in the morphology of worm-like aggregates. The morphology of surfactant aggregates is a function of the degree of curvature of either the surfactant monolayer or bilayer. For hydrocarbon-based surfactants, the curvature of the aggregate is strongly influenced by the packing parameter *p*, *p* = *v*/*al.* Where *v* is the hydrophobic volume, *l* is the hydrophobic chain length of the surfactant molecule, and *a* is the effective cross-sectional area of the hydrophilic group. In general, micelles are spherical for *p* is less than 1/3. When *p* is between 1/3 and 1/2, worm-like micelles are expected. When *p* is between 1/2 and 1, lamellar structures should be formed spontaneously. At low concentrations, 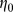 remains almost unchanged, which is due to the formation of a large number of spherical micelles in the solution. As the concentration of SL increased, the lauric anions bind strongly to the cations and greatly reduce the repulsion between the cations, thus significantly reduce *a*. Thus, addition of SL to the micellar solution increase the packing parameter *p*, long micelle entities and transient entangled networks that consist of the entities were formed in the solutions, which gave rise to the maximum 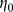. The decrease in 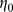 is generally considered to be the shortening of contour length of micelles. It is generally recognized that, when *p* increases to a certain degree that provides favorable conditions for forming highly curved endcaps to help to shorten the length of micelles [17].

### Rheological model of worm-like micelles

**Figure 3:**
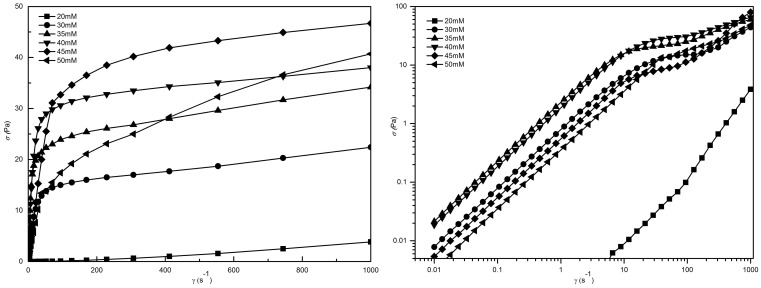
Shear stress with different concentration of SL at a fixed C_16_MDB concentration of 70 mM.

The shear stress versus shear rate of C_16_MDB at 70 mM in the presence of SL at all the studied concentrations is shown in Figure 3a. Shear stress increases linearly with shear rate, indicating that the 70 mM C_16_MDB/20 mM SL sample exhibits the characteristics of a Newtonian fluid. This result is consistent with the results of the steady shear measurement. When the concentrations of SL are greater than 20 mM, apparent yield stress values become important and reach a maximum as concentrations of SL reach 45 mM. Thus, these samples show pseudo-plastic fluid features. Figure 3b displays the log-log plot of C_16_MDB at 70 mM in the presence of SL at all the studied concentrations, and there is a plateau of shear stress that is incorrectly identified as the yield stress value at shear rates between approximately 10 and 100 Hz when the concentrations of SL are greater than 20 mM. However, the stress and shear rates showed a linear relationship overall. Therefore, one can draw a conclusion that all of samples do not have a true yield stress value in effect.

### The viscoelasticity of worm-like micelles

**Figure 4:**
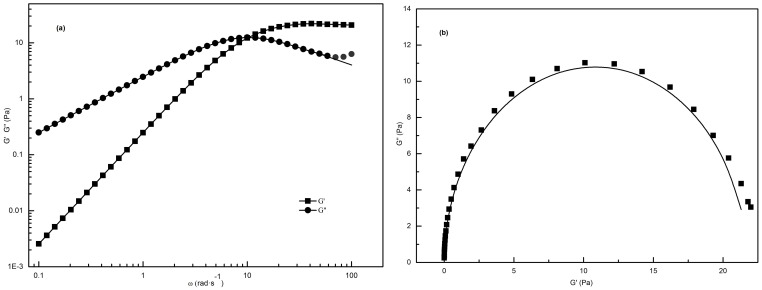
Dynamic oscillatory plots. (a) Variations of G′ and G″ as a function of frequency (*ω*) in aqueous 70 mM C_16_MDB/35 mM SL solution; (b) Cole–Cole plots (solid lines indicate the best fitting of Maxwell model).

Figure 4a demonstrates the variation in the storage modulus G′ and loss modulus G″ as a function of frequency *ω*. In the low frequency region, the systems showed a liquid-like behavior (G′<G″). In the high frequency region, the systems showed solid-like behavior (G′>G″). In addition, there is one intersection point between G′ and G″ at a specific frequency 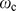, which is dependent on the concentration of SL. The solid lines represent the best fit of a Maxwell model to the experimental data. The data are well fit by the Maxwell Model in the range of low and medium frequencies, which is illustrated in Figure 4a. The small deviations at high frequencies are another feature of worm-like micelles, which is due to the worm-like micelles undergoing a dynamic equilibrium with rapid breaking and recombination [18].

By calculating the deviation of the data points from the Maxwell model, a “Cole-Cole” plot can estimate how well the data fits Maxwell model. The “Cole-Cole” plot of *G″* as a function of *G′* is described as [13]:
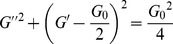
(3)


Figure 4b shows that the system is well fit the Maxwell model at low and medium frequency, whereas the degree of deviation from the semicircular behavior at high frequency can be obtained by measuring the deviation of the data points.

Surfactant solutions with network structures, such as worm-like micelles, are consistent with the Cox-Merz rule, which states that the steady-shear viscosity (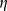) and the complex viscosity (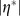) are equal when shear rate (*γ*) and frequency of oscillation (*ω*) are equal according to the following equations [19], [20]:
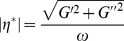
(4)

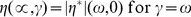
(5)


**Figure 5:**
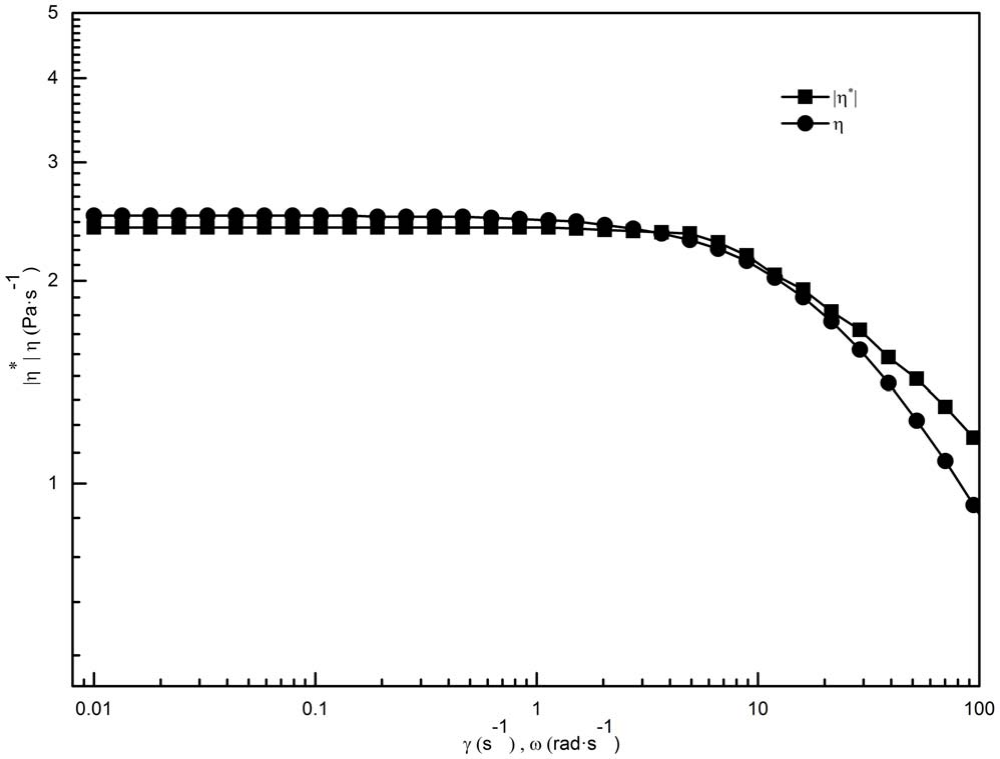
Shear-rate dependence of the steady-shear viscosity and frequency dependence of the complex viscosity for the 70 mM C_16_MDB/35 mM SL solution.

As shown in Figure 5, the coincidence of 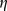 and 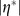 indicates that worm-like micelles that possess a rigid network structure have already formed in the 70 mM C_16_MDB/35 mM SL solution. The formation of branched network of micelles, polydispersity in relaxation time, reversible scission of micelles in addition to involvement of breathing modes are possible reasons for the deviation occurs in the high frequency region [17].

The rheological parameters related to the microstructures of C_16_MDB solutions with different SL concentrations have been calculated according to the following formulas [1], and the results are listed in Table 1.
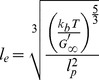
(6)

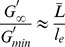
(7)Here, *l_e_* is the entanglement length, *l_p_* is the persistence length and usually varies with surfactant structure, counter-ion and salt concentration. In order to simplify the calculation, we assume that the persistence length *l_p_* of the micelles is a constant and set *l_p_* = 15 nm according to the literature [21], *T* is the absolute temperature, 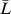 is the average contour length, and *k_b_* is the Boltzmann constant. From Table 1, it can be observed that 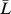 is largest and *ω_c_* is the smallest for the sample at 35 mM SL. The 70 mM C_16_MDB/35 mM SL solution has the longest worm-like micelles and the longest kinetic lifetimes. At the same time, it should be noted that the average contour length of the micelles decreased monotonously after reaching the maximum at 35 mM SL. The results proved that the shortening of contour length of the micelles led to the decrease of 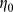, in accordance well with the steady-state shearing measurements.

**Table 1:** Various rheological parameters calculated for the aqueous solutions with different SL concentration at a fixed concentration of 70 mM C_16_MDB.

SL(mM)	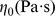	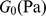	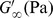	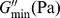	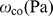	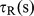	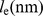	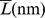
30	0.79	8.50	12.17	6.83	18.94	0.053	194	346
35	2.19	21.03	24.90	4.84	10.29	0.097	130	670
40	1.84	29.41	36.60	9.24	20.11	0.049	105	417
45	0.54	14.78	19.12	36.3	35.37	0.028	87	79
50	0.22	3.51	4.32	31.8	39.64	0.024	69	45

### The effect of temperature

**Figure 6:**
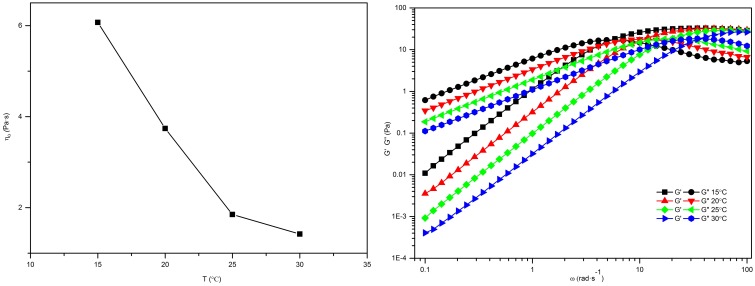
Temperature effect plots. (a) The zero shear viscosity 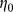 as a function of temperature for the 70 mM C_16_MDB/40 mM SL solution; (b) the frequency sweep of 70 mM C_16_MDB/40 mM SL system at different temperatures.

We also studied whether temperature has an impact on the formation of worm-like micelles. Generally, a worm-like micelle solution is temperature sensitive, in which physicochemical changes resulting from small external variations in environmental conditions matter. To show this effect more quantitatively, steady and dynamic rheological measurements were carried out on 70 mM C_16_MDB/40 mM SL solution sample. The temperature dependent experiments in this work were limited to 15–30°C, and the related rheological parameters are calculated in Table 2. The zero shear viscosity 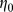 as a function of temperature is depicted in Figure 6a. With increasing temperature, the 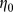 decreased monotonically. Figure 6b presents the frequency sweep of 70 mM C_16_MDB/40 mM SL system at different temperatures. With increasing temperature, the entire frequency curve shifts to the right, and the crossover frequency 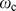 becomes correspondingly larger, which leads to shorter relaxation times. At the same time, though, we noticed that the constant *G_0_* which indicates the entanglement degree of the micellar structure, remained about the same while the micellar contour length 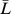 decreased exponentially with an increase of temperature. The viscosity decreases, the relaxation time shortens, and *G_0_* remains unchanged upon heating in the 70 mM C_16_MDB/40 mM SL system; these observations could be attributed to decreases in the micellar contour length 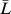 and could account for the more rapid hopping of surfactant molecules at higher temperatures [22].

**Table 2:** Various rheological parameters calculated for the sample of 70 mM C_16_MDB/40 mM SL at different temperatures.

*T*(°C)	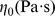	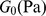	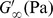	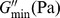	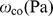	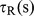	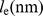	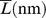
15	6.07	32.42	34.11	4.98	5.49	0.18	107	735
20	3.74	31.97	35.58	6.63	10.76	0.09	106	568
25	1.85	30.65	36.60	9.24	20.11	0.05	105	417
30	1.42	30.26	36.72	12.30	36.05	0.03	105	316

**Figure 7:**
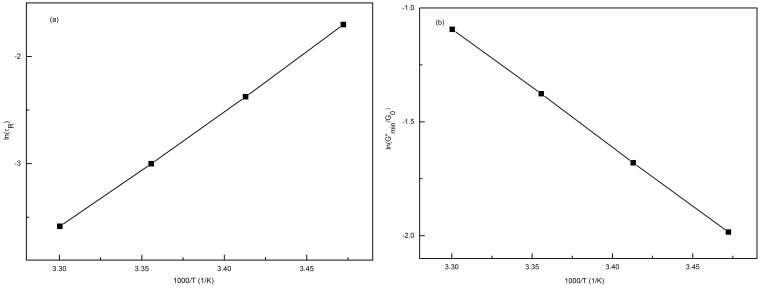
Energy calculation plots. (a) An Arrhenius plot of ln*τ*_R_ as a function of 1/T for the 70 mM C_16_MDB/40 mM SL solution; (b) the plot of ln (G″_min_/G_0_) as a function of 1/T.

The value of *E_a_* and *E_scis_* can be obtained from an Arrhenius plot of ln 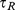 versus 10^3^/*T* as given by [23], [24]:
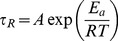
(8)

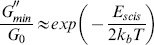
(9)Here, *E_a_* is the flow activation energy, which is necessary to move individual micelles in an environment of surrounding micelles. *E_a_* should be proportional to 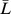 because of the definitions of *E_a_* and 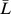. *E_scis_* is the scission energy, which is required to create two end-caps from a semi-infinite cylinder and is an exponential function of 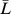. *R* is the gas constant, and *A* is a constant. The value of *E_a_* calculated from the slope in Figure 7a is approximately 91.05 kJ mol^−1^ for the 70 mM C_16_MDB/40 mM SL solution. Figure 7b presents the plot of ln (*G″_min_*/*G_0_*) as a function of the reciprocal of the absolute temperature. The value of *E_scis_* calculated from the slope in Figure 7b is equal to 86.13 kJ mol^−1^, which is close to the values of conventional ionic surfactants reported previously [24], [25].

### Cryo-transmission electron microscopy

**Figure 8:**
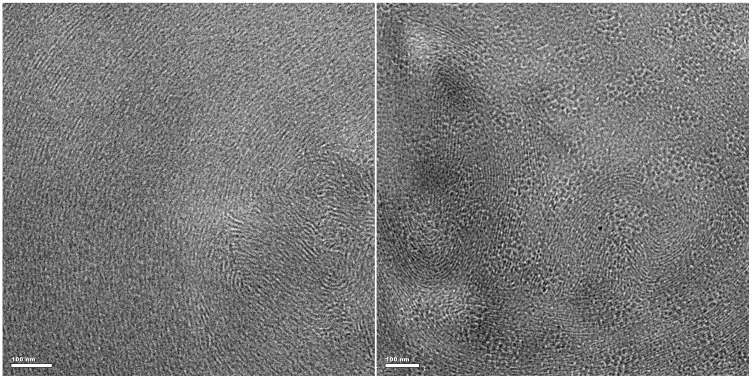
Cryo-TEM images. (a) Cryo-TEM image of the 70 mM C_16_MDB/35 mM SL solution; (b) Cryo-TEM image of the 70 mM C_16_MDB/40 mM SL solution.

For the moment, the cryo-TEM technique is the best and most direct method to characterize worm-like micelles, as it can provide the most powerful evidence to the formation and direct morphology of worm-like micelles in solution without staining- or drying-associated artifacts [26]. Figures 8a and 8b show that a large number of worm-like micelles are present in the 70 mM C_16_MDB/35 mM SL solution and 70 mM C_16_MDB/40 mM SL solution, respectively. Moreover, the micelles are found to entangle into three-dimensional network structures by overlapping each other. Because it is difficult to identify the beginning and end of entangled worm-like micelles, the specific contour length of worm-like micelles could not be obtained.

## Conclusions

In summary, with the aid of sodium laurate, the aggregation behavior of worm-like micelles in aqueous solution was observed using *N*-hexadecyl-*N*-methylpiperidinium bromide as the cationic surfactant. Rheological measurements revealed that the main factors affecting the viscoelastic properties of worm-like micelles are temperature and the concentrations of sodium laurate. The scission recombination mechanism combined with the higher diffusion rate of the cylindrical part of the micelles leads to pseudoplastic behavior and shear thinning in the steady shear rheology experiments. For the C_16_MDB/SL mixed solutions, the estimated values of several important rheological parameters as functions of the solution composition and temperature are calculated. At the same time, the flow activation energy and the scission energy of worm-like micelles formed in 70 mM C_16_MDB/40 mM SL solution were compared with other published results. Cryo-TEM imaging visually observed the formation of entangled micelles, which supports the rheological experimental results. We believe that this work has further explained the mechanism of formation of worm-like micelles with high viscoelasticity and serves as a possible application of ILs in colloidal systems.
